# Lichenoid mucocutaneous reactions associated with sintilimab therapy in a non-small cell lung adenocarcinoma patient: case report and review

**DOI:** 10.3389/fphar.2023.1276788

**Published:** 2023-12-12

**Authors:** Shuting Zhou, Zhenyu Zhang, Xiaodong Feng, Chengjian Zhao, Lu Jiang

**Affiliations:** ^1^ State Key Laboratory of Oral Diseases, National Center for Stomatology, National Clinical Research Center for Oral Diseases, Chinese Academy of Medical Sciences Research Unit of Oral Carcinogenesis and Management, Department of Oral Medicine, West China Hospital of Stomatology, Sichuan University, Chengdu, Sichuan, China; ^2^ State Key Laboratory of Biotherapy and Cancer Center, West China Hospital, Sichuan University, and Collaborative Innovation Center for Biotherapy, Chengdu, Sichuan, China

**Keywords:** lichenoid reactions, Sintilimab Therapy, immune checkpoint inhibitors (ICIs), PD-1 inhibitor, mucocutaneous reactions, immune-related adverse events (irAEs), non-small cell lung adenocarcinoma

## Abstract

The immune checkpoint inhibitor (ICI), anti-programmed cell death receptor-1 (PD-1) antibody, has gained widespread approval for treating various malignancies. Among the immune-related adverse reactions (irAEs) during ICI treatment, the lichenoid reaction is noteworthy. Sintilimab, a new PD-1 inhibitor, has secured approval in China for treating refractory non-Hodgkin’s lymphoma, and phase I/II clinical trials for other solid tumors are ongoing both domestically and abroad. This paper presents a case of a mucocutaneous lichenoid reaction associated with sintilimab therapy, its diagnosis, and management. Our study, using multiplex immunofluorescence staining, reveals localized infiltration of CD4^+^ and CD8^+^ T lymphocytes in the subepithelial lamina propria region with upregulated PD-1 expression, implying an association between PD-1 expression upregulation and lichenoid reactions provoked by PD-1 monoclonal antibody. We provide a summary of clinical characteristics and treatment guidelines for lichenoid reactions induced by ICIs from previous reports, highlighting the success of a combined therapeutic regimen of oral antihistamines and topical corticosteroids in controlling symptoms without interrupting ICI treatment.

## 1 Introduction

Lung cancer remains the leading cause of cancer-related mortality worldwide. The standard treatment protocol involves multimodal therapy, encompassing radiation, chemotherapy, and surgery. Recent advancements, particularly in combination immunotherapy, have significantly improved the 5-year survival rates (Herbst et al., 2022). Additionally, various plant and herbal extracts have demonstrated anticancer effects across multiple cancer cell types (Pathiranage et al., 2020).

At the heart of these advancements are immune checkpoints, a complex array of inhibitory pathways integral to the immune system. These pathways play crucial roles in maintaining self-tolerance and modulating immune responses to prevent collateral tissue damage (Pardoll, 2012). They regulate T-cell activity against tumor cells through mechanisms such as co-inhibition or co-stimulation of signaling pathways. Immune checkpoint inhibitors (ICIs), including agents targeting CTLA-4, PD-1, and PD-L1, have revolutionized the treatment of multiple malignancies (Postow et al., 2018). However, ICIs can also amplify nonspecific immune activities, leading to immune-related adverse events (irAEs) affecting various systems, including dermatologic and mucosal systems (Postow, 2015; Sibaud et al., 2016).

Reports of mucosal involvement in PD-1 and PD-L1 blockade therapies, although sporadic, have highlighted a range of manifestations from lichenoid lesions to Steven Johnson Syndrome/Toxic Epidermal Necrolysis (Saw et al., 2017; Sibaud et al., 2017; Obara et al., 2018; Utsunomiya et al., 2018; Senoo et al., 2020; Shazib et al., 2020; Costedoat et al., 2021). Sintilimab, a fully human IgG4 monoclonal antibody against PD-1, approved by the US FDA and China NMPA for certain cancer treatments, has been implicated in such reactions (Hoy, 2019; Zhang et al., 2022).

This paper presents a novel case of a non-small cell lung adenocarcinoma patient who developed lichenoid reactions following sintilimab therapy. This is, to our knowledge, the first reported instance of lichenoid reaction associated with sintilimab. We examine the underlying mechanisms of this reaction, based on both literature review and detailed analysis of our case. We also propose clinical management strategies for such irAEs, based on the insights gained from this case study. This report aims to contribute to the growing body of knowledge on ICIs’ dermatologic and mucosal side effects, emphasizing the importance of case studies in understanding and managing these complex reactions.

## 2 Case presentation

A 38-year-old male patient reported oral mucosa discomfort persisting for 2 weeks to our hospital. Three years prior, the patient had undergone a right lower lung lobectomy due to a diagnosis of non-small cell lung adenocarcinoma. Two years ago, the patient commenced treatment with Camrelizumab, a programmed cell death inhibitor, at a dosage of 200 mg intravenously every 2 weeks (Markham and Keam, 2019). Following the initial injection, the patient developed acute pancreatitis and hyperglycemia. After two additional doses, ketoacidosis ensued, and a diagnosis of type I diabetes was made. Consequently, PD-1 inhibitor therapy was discontinued, and insulin was administered for glycemic control. The patient subsequently received treatment with docetaxel and began a regimen of the PD-1 inhibitor sintilimab every 3 weeks, commencing 3 months ago. After three treatment cycles, the patient developed pruritic skin papules on the palms and soles of the feet. These lesions were marginally improved through the use of rupatadine fumarate tablets and loratadine tablets. Following the fourth sintilimab cycle, the patient reported inner lip irritation and pain when consuming spicy or hot foods, but the skin lesions did not recur. The patient had no history of other systemic diseases or allergies and had no family history of genetic disorders.

**FIGURE 1:**
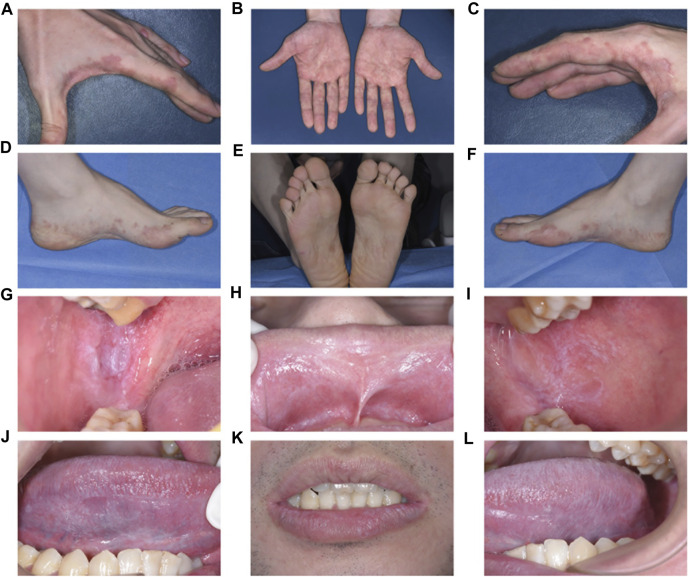
Clinical examination and Intraoral examination **(A–F)**. Irregular pink to purplish flat-topped papules and plaques with hyperpigmentation on the palms and dorsum of the hands and soles of the feet **(G–L)**. Symmetrical papular white streaks with mild erosions and congestion on the oral mucosa and the inside of the upper and lower lips, and reticular white streaks consistent with Wickham’s streaks could be seen on the ventral part of the tongue and lips bilaterally.

Clinical examination exposed irregular pink to purplish flat-topped papules and plaques with hyperpigmentation on the palms, dorsum of the hands, and soles of the feet (Figures 1A–F). Intraoral examination revealed symmetrical papular white streaks with mild erosions and congestion on the oral mucosa and the inner surface of the upper and lower lips. Reticular white streaks, consistent with Wickham’s streaks, were observed on the ventral part of the tongue and lips bilaterally (Figures 1G–L).

All routine blood tests, biochemistry analyses, erythrocyte sedimentation rates, coagulation tests, and infection tests yielded negative results. The anti-desmoglein-1 antibody, anti-desmoglein-3 antibody, and anti-BP180 antibody were all negative.

**FIGURE 2:**
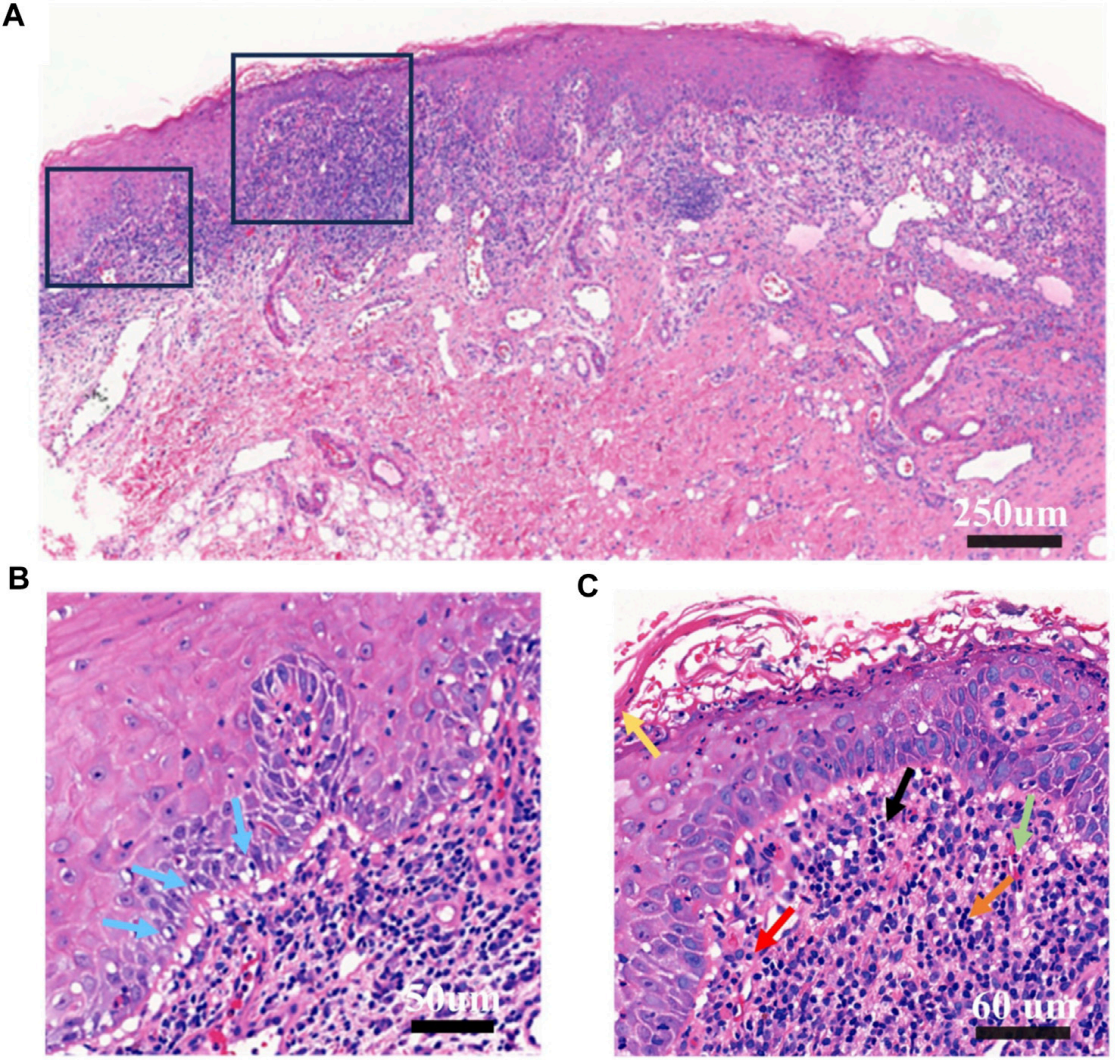
Histopathological examination of buccal mucosa **(A)**. Original magnification at ×10 Chronic inflammation; hyperkeratosis of epithelial surface layer; vacuolar degeneration and liquefaction degeneration of some basal cells; capillary proliferation and expansion of lamina propria, more eosinophils, neutrophils, lymphocytes, plasma cells infiltration; proliferation and degeneration of fibrous tissue **(B)**. Original magnification at ×20. This panel highlights vacuolar and liquefaction degeneration of select basal cells, indicated by blue arrows. **(C)** Original magnification at ×40. The panel features extensive infiltration by eosinophils, neutrophils, lymphocytes, and plasma cells. Yellow arrow points to epithelial hyperkeratosis; the green arrow denotes eosinophils; the black arrow indicates neutrophils; the orange arrow designates lymphocytes; and the red arrow identifies plasma cells.

A biopsy was performed on the oral buccal mucosal lesion. Histopathological examination (Figures 2A–C) and IgA, IgG, IgM, and C3 direct immunofluorescence tests yielded negative findings, which indicated a lichenoid lesion. Incorporating the patient’s history, clinical examination, laboratory tests, and histopathological examination, a diagnosis of an oral lichenoid reaction was confirmed.

**FIGURE 3:**
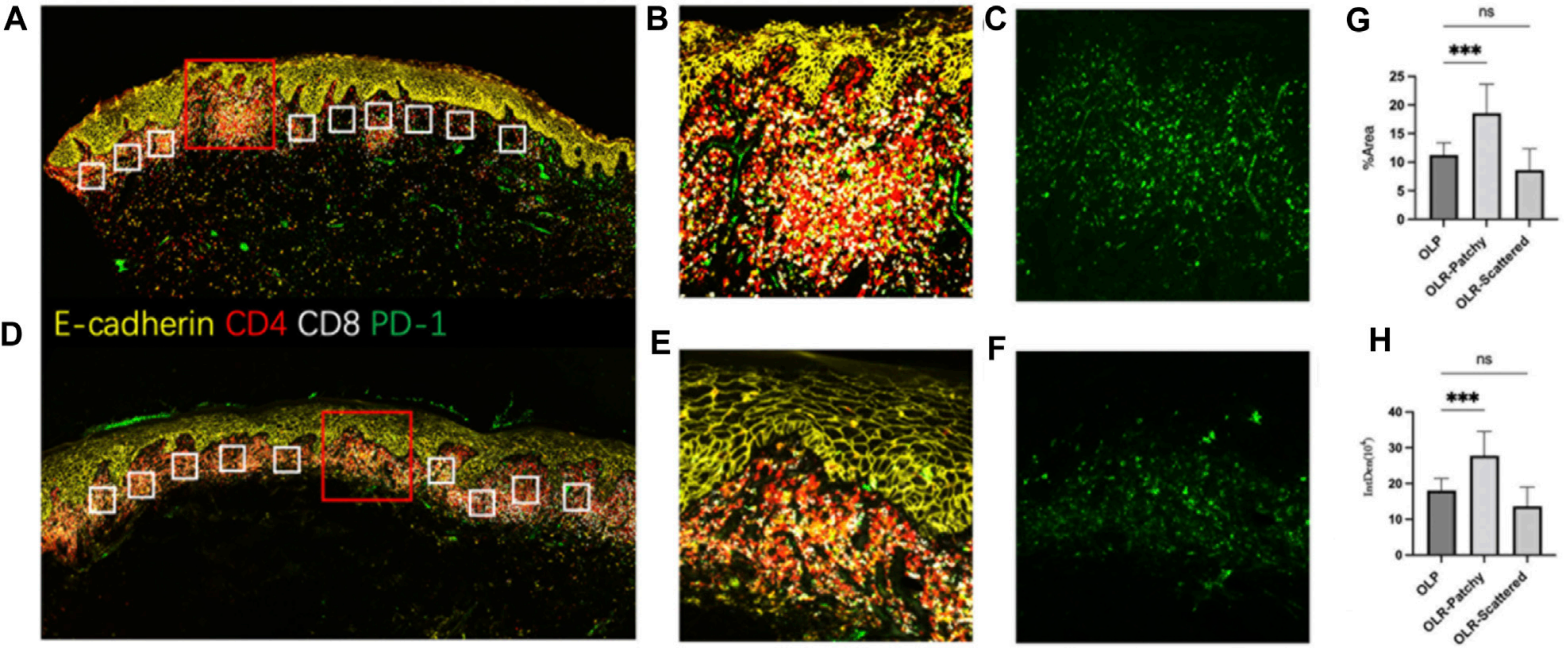
The multiplex immunofluorescence staining **(A–C)**. The antigen distribution pattern observed in OLR. **(D–F)**. The antigen distribution pattern observed in OLP. **(G,H)** Ten areas of equal size were randomly selected from each of the three groups for statistical analysis, including focal infiltration of inflammatory cells in OLR, scattered infiltration of inflammatory cells in OLR and OLP. **(G)**: Comparison of the area% of PD-1 fluorescence-positive expression in OLR and OLP, *p* < 0.001. **(H)**: Comparison of the integrated density of PD-1 fluorescence-positive expression in OLR and OLP, *p* <0.001 Note: **(A–F)** Staining using Luminlris™ HyperView multiplex immunostaining kit (IRISKit™MH010101). Primary antibodies were mouse anti-PD-1(ZSGB-BIO, ZM-0381), mouse anti-CD8(Immunoway, YM6938), and rabbit anti-CD4(HUABIO, ET1609-52).

Further analysis was conducted on the distribution of lymphocyte subtypes and associated biomarkers, including CD4, CD8, and PD-1. This was achieved utilizing the Luminlris™ HyperView multiplex immunostaining kit (IRISKit™MH010101), as shown in Figures 3A–F. Although a banded distribution of lymphocyte infiltration is typically seen in oral lichen planus, our case exhibited a pattern of continuous rather than banded infiltration. The distribution of PD-1 on lymphocytes was evaluated and classified into two zones of patchy and scattered distribution, alongside typical oral lichen planus lesions. Focal infiltration by inflammatory cells was found to coincide with an upregulation in PD-1 expression. Notably, PD-1 expression in the patchy area of inflammatory cells was higher than that in oral lichen planus lesions, while its expression in scattered areas showed no significant differences (Figures 3G, H).

Following a few days of gargling with a dexamethasone sodium phosphate solution, the patient’s symptoms markedly improved. After 1 month of usage, the majority of the oral lesions began to diminish. Although oral mucosal lesions reappeared regularly during subsequent sintilimab treatments, the skin lesions did not. The oral mucosal symptoms were effectively managed with a dexamethasone sodium phosphate gargle. Following the eighth sintilimab injection, the patient developed hypothermia and experienced worsened oral mucosal symptoms with more extensive and longer-lasting lesions than before. These symptoms were effectively managed with a topical dexamethasone sodium phosphate solution gargle and triamcinolone acetonide dental paste. In accordance with the oncologist’s advice and the patient’s preference, sintilimab treatment was discontinued after the completion of the eighth cycle. Subsequent follow-up assessments were conducted at 3- and 6-month intervals post-discontinuation, revealing no recurrence of intraoral lichenoid reactions.

## 3 Discussion

This study discusses the unique case of a patient with non-small cell lung adenocarcinoma who developed lichenoid lesions after treatment with sintilimab, a phenomenon not previously reported. The characteristic symptoms of immune-related adverse events (irAEs), such as fever, diarrhea, and organ damage, including skin damage, are well-documented in the use of sintilimab (Li et al., 2021; Huang et al., 2022). However, the present case adds to the understanding of potential adverse reactions to this treatment.

The lichenoid lesions observed in our patient differed from traditional oral lichen planus in several key ways. The lesions exhibited reticular white streaks akin to Wickham’s streaks, along with continuous, but not band-like, lymphocyte infiltration adjacent to the basal cell layer. These lesions also presented with a greater infiltration of eosinophils, neutrophils, and plasma cells. The patient responded positively to the administration of topical glucocorticoids, such as dexamethasone and triamcinolone acetonide, which enabled the continuation of sintilimab treatment without interruption.

Oral lichen reactions warrant differentiation from oral lichen planus (OLP) and other lichenoid lesions that may be indicative of bullous diseases. Both direct immunofluorescence and ELISA techniques were employed to rule out bullous conditions (Van Der Meij and Van Der Waal, 2003). The updated diagnostic criteria for OLP, modified from WHO 2003 criteria, were delineated in 2016 (Louisy et al., 2023). These criteria define characteristic clinical features of OLP as well-defined, looping, and intersecting white striae against a backdrop of variable erythema. The lesions typically exhibit a roughly symmetrical distribution. Microscopically, OLP is characterized by hyperparakeratosis, hyperorthokeratosis, or a combination of both, along with cytoid (Civatte) bodies, basal cell hydropic change, and a predominantly lymphocytic infiltrate in the lamina propria (Cheng et al., 2016). In cases where these microscopic features are not clearly exhibited, the term “histopathologically compatible with OLP” is proposed. Under these revised criteria, a definitive diagnosis of OLP necessitates both clinical and histopathological features to be in complete alignment. Any deviation leads to a diagnosis of either oral lichenoid lesion (OLL) or oral lichenoid reaction (OLR) (Cheng et al., 2016). In the present case, clinical manifestations included reticular white patterns in the oral mucosa, while histopathological examination revealed vacuolar and liquefactive degeneration of basal cells, accompanied by infiltration of lymphocytes, eosinophils, and plasma cells in the lamina propria. The patient’s medical history did not indicate mucocutaneous lesions during 2 years of docetaxel treatment, an agent known to interfere with microtubule activity during cell cycle progression (Gupta et al., 2023). Furthermore, a cross-sectional study demonstrated minimal incidence of lichenoid reactions related to chemotherapy (Jena et al., 2022). Interestingly, immune checkpoint inhibitors similar to sintilimab, such as pembrolizumab and nivolumab, have been associated with mucocutaneous lichenoid reactions. In light of the patient’s clinical history and the histopathological findings, there is a strong implication that sintilimab played a pivotal role in the development of oral lichenoid lesions. Consequently, we propose the hypothesis that the oral lichenoid reaction observed in this patient was primarily induced by sintilimab therapy.

The exact pathophysiological mechanisms behind drug eruptions induced by PD-1 monoclonal antibodies have yet to be comprehensively elucidated. Several potential mechanisms are hypothesized, encompassing enhanced T-cell activity against antigens residing in both tumors and healthy tissue, augmentation of pre-existing autoantibodies, and an escalated level of inflammatory cytokines (Postow et al., 2018). The PD-1-PD-L pathway, comprising PD-1 and its ligands PD-L1 (B7-H1) and PD-L2 (B7-DC), has been implicated in these processes. Regarded as a crucial negative regulator of T-cell activation, PD-1 (Okazaki and Honjo, 2006) transmits inhibitory signals that modulate T-cell proliferation, cytokine production, and cytolytic function upon binding with PD-L1 or PD-L2 (Riley, 2009). The blockade of PD-1 or PD-L1 leads to enhanced T-cell activation, consequently inhibiting tumor development. Interestingly, PD-1 inhibitors have been demonstrated to shift antigen-induced cellular reactivity toward a pro-inflammatory Th1/Th17 response. This effect was denoted by the heightened expression of Th1 and Th17 associated markers like interferon γ, interleukin (IL)-2, tumor necrosis factor α, IL-6, and IL-17, while simultaneously suppressing the production of IL-5 and IL-13 cytokines linked to Th2 (Dulos et al., 2012). The B7-H1 blockade also significantly increased the production of IFN-γ and IL-2, akin to the response observed with anti-PD-1 (Zhou et al., 2012). An imbalance in the microenvironmental cytokine profile is closely associated with the onset of lichenoid reactions. The pathogenesis of such reactions is believed to be initiated by the attack and destruction of the basal epidermal layer by autoreactive cytotoxic T cells secreting a TH1-type cytokine profile (Meller et al., 2009). Th1 pro-inflammatory immune response predominance is typically observed in lichenoid dermatitis—irAE (Curry et al., 2019). The involvement of T cells in the pathogenesis of oral lichen planus (OLP) has been extensively studied. Initial evidence for the crucial role of T cells came from a study in which a cytotoxic CD4^+^ T-cell clone was found to induce oral lesions following localized metastases (Shiohara et al., 1986). Subsequent research has pointed towards cytotoxic CD8^+^ T cells as primary agents in inducing apoptosis in keratinized cells (Sugerman et al., 2000). This T-cell-mediated etiology of OLP was further refined in 2016 (Wang et al., 2016). Programmed cell death protein 1 (PD-1) is ubiquitously expressed in tumor-infiltrating lymphocytes (TILs) across various tumor types. Elevated PD-1 expression in CD4^+^ TILs often mirrors heightened PD-1 levels in regulatory T cells (Treg), while an increase in PD-1 expression on CD8^+^ TILs could indicate anergic or exhausted states (Pardoll, 2012; Farhood et al., 2019). In the context of NSCLC, higher PD-1 levels have been observed on lymphocytes in metastatic lymph nodes (Ma et al., 2017). In the current case, we postulate that the administration of a PD-1 monoclonal antibody led to enhanced local lymphocyte infiltration in the epithelial submucosa, subsequently resulting in mucosal lichenoid lesions. In our investigation, we specifically aimed to evaluate the distribution patterns of CD4^+^ and CD8^+^ T cells in oral lichen-like lesions elicited by sintilimab. Our intent was to determine whether these patterns deviate from those documented in prior literature. The observed distribution profiles of CD4^+^ and CD8^+^ T cells in the present case not only reinforce the diagnosis of a lichenoid reaction but also align well with previously published studies. Concurrently, PD-1 expression was examined to elucidate the underlying mechanisms contributing to lichenoid reactions caused by anti-PD-1 therapy. Consistent with this, in the present case study, high PD-1 expression was noted in regions harboring inflammatory cells. The enhanced expression and activation of PD-1 negatively regulate T lymphocytes, which are crucial for maintaining immune tolerance in normal organisms and contribute to tumor immune evasion during tumorigenesis. Meanwhile, PD-1 monoclonal antibody heightens T-cell activation by blocking the PD-1/PD-L1 signaling pathway. In this specific case, localized CD4^+^ and CD8^+^ T-cell infiltration was observed along with increased PD-1 expression in the subepithelial lamina propria (Figure 3). Based on these findings, we propose that the mucosal lichenoid reaction observed in our patient might be associated with the increased expression of PD-1 in NSCLC patients. The administration of PD-1 antibodies to inhibit receptor binding could result in heightened focal T-cell expression, further leading to the emergence of adverse mucosal reactions. Interestingly, cessation of sintilimab use led to the disappearance of the mucosal adverse effects, substantiating our hypothesis. Further studies are warranted to validate this proposed mechanism and explore its clinical implications.

Immune checkpoint inhibitors (ICIs) can result in adverse effects that inconvenience patients, reduce their quality of life, or, in extreme cases, necessitate the suspension or termination of therapy. However, these adverse events also indicate that ICIs are promoting effective immune activation in patients. Notably, some studies have reported an association between the frequency of adverse events and the efficacy of immunotherapy, suggesting that patients with cutaneous adverse reactions may have a lower risk of tumor progression (Chan et al., 2020; España et al., 2020).

We summarize previously reported cases of mucocutaneous lichenoid reactions to anti-PD-1/PD-L1 agents with well-documented records (Shi et al., 2016; Sibaud et al., 2017; Obara et al., 2018; Enomoto et al., 2019; Kan et al., 2021; Niesert et al., 2021), indicated that most reactions are manageable, allowing continued use of immune checkpoint inhibitors (Table 1). The management of such adverse reactions typically involves the use of topical steroids, with more severe cases requiring oral antihistamines and corticosteroids, or even discontinuation of PD-1 inhibitor therapy.

**TABLE 1:** Mucocutaneous Lichenoid Reactions Induced by Anti-PD-1/PD-L1 Inhibitors: A review of reported cases.

Inhibitor utilized	Sex/Age	Underlying malignancy	Treatment cycle	Oral clinical manifestations	Concurrent dermatological lesions	Mucosal histopathological pattern	Cutaneous histopathological pattern	Management strategy	Patient outcome	Therapy discontinuation due to rash
Nivolumab										
Shi et al.	F/NA	RCC	2	Reticular lesions	Papular (palms、soles)	Lichenoid mucositis	NR	Clobetasol, PUVA	Complete Resolution	Y
Shi et al.	F/NA	Lung Cancer	14	Erosive lesions	None	Lichenoid mucositis	NR	Clobetasol, Valacyclovir	Complete Resolution	Y
Sibaud et al.	M/NA	Metastatic melanoma	1	Erosive lesions	Erosive genital lichen planus	Lichenoid mucositis	NR	Clobetasol	Complete Resolution	N
Sibaud et al.	M/53	Multiple myeloma	2	Reticular lesions	Cutaneous lichenoid eruption, with genital involvement	NR	NR	Topical dexamethasone	Complete Resolution	N
Sibaud et al.	M/62	RCC	23	Reticular lesions; Plaque-like lesions	None	①②③	NR	None	Resolution Following Drug Discontinuation	Y
Sibaud et al	M/42	GBM	2	Reticular lesions	None	NR	NR	Topical corticosteroids; Anti-fungal lozenges	NR	NR
Sibaud et al.	F/70	ENMZL (Lung)	6	Reticular lesions; Atrophic lesions	Cutaneous lichenoid eruption	NR	Lichenoid interface dermatitis	Topical and oral corticosteroids	Complete Resolution	N
Sibaud et al.	M/63	Lung carcinoma	3	Reticular lesions	Nonspecific maculopapular rash	NR	NR	None	NR	NR
Enomoto et al.	M/52	LUAD	10	Erosive lesions	None	①②	NR	Oral prednisolone 1.0 mg/kg/d	Complete Resolution	Y
Obara et al.	M/67	LUAD	2	Ulcerative lesions	None	①②	NR	Topical triamcinolone	Complete Resolution	Y
Obara et al.	F/74	LUAD	7	Ulcerative lesions	Erythematous papules with scales (hands and trunk)	①②	Lichenoid interface dermatitis	Oral prednisolone 60 mg/d	Complete Resolution	Y
Pembrolizumab										
Sibaud et al.	F/41	Breast cancer	10	Reticular lesions	None	①②③	NR	None	NR	NR
Sibaud et al.	M/58	Lung carcinoma	12	Reticular lesions	Diffuse cutaneous lichenoid eruption, with genital involvement	NR	NR	Topical corticosteroids	Complete Resolution	N
Niesert et al	M/77	Malignant melanoma	8	Reticular lesions	Multiple nodules and plaques (both lower legs)	①②	NR	Topical steroids; Keratolytic ointment; Oral prednisolone 1.0 mg/kg/d	Complete Resolution	N
Atezolizumab										
Shi et al.	F/NA	RCC	11	Erosive lesions	Papular (palms, arms)	Lichenoid mucositis	NR	Topical clobetasol; NB-UVB	Complete Resolution	Y
Sibaud et al.	M/56	RCC	11	Plaque-like lesions; Reticular lesions	Cutaneous lichenoid lesion with nail involvement	①②③	①②③	Topical corticosteroids	Resolution Following Drug Discontinuation	Y
Sibaud et al.	M/66	EAC	14	Reticular lesions	None	①③	NR	None	NR	NR
Sibaud et al.	M/54	RCC	5	Ulcerative lesions	None	NR	NR	Topical corticosteroids	Complete Resolution	N
Toripalimab										
Kan et al.	M/78	Metastatic prostate cancer	5	Ulcerative lesions	Plaques (lower limb)	①②	NR	Oral prednisolone 15 mg/d; Helium–neon laser	Complete Resolution	N
Sintilimab										
Our case	M/38	NSCLA	4	Reticular lesions	Papular (palms, soles)	②③③⑤	NR	Topical dexamethasone	Complete Resolution	N

F, female; M, male; NA, not available; NR, not reported.

RCC, renal cell carcinoma; GBM, glioblastoma multiforme; ENMZL, extranodal marginal zone lymphoma; LUAD, lung adenocarcinoma; ECA, esophageal adenocarcinoma; NSCLA, Non-small cell lung adenocarcinoma.

NB-UVB, Narrow-band ultraviolet B; PUVA, Psoralen plus ultraviolet A; BMZ, basement membrane zone.

① Band-like lymphocytic infiltrate in the upper lamina propria; ② Partial disruption of the BMZ; ③ Hypergranulosis and parakeratosis; ④ Not band-like lymphocytic infiltrate in the upper lamina propria; ⑤ The infiltration is mixed with plasma cells and eosinophils.

In conclusion, our findings suggest that irAEs induced by ICIs, including sintilimab, can be effectively managed through topical glucocorticoid administration. For severe cases, the combination of oral antihistamines or glucocorticoids may be considered without necessitating drug discontinuation.

As the use of ICIs in cancer treatment expands, the occurrence of irAEs will likely increase. Hence, it is crucial to develop effective strategies for managing irAEs, allowing patients to continue ICI therapy. These strategies should be tailored to each patient’s unique circumstances, taking into account factors such as the severity of irAEs, future cancer treatment plans, and tumor response to ICIs, balancing the risks and benefits of irAE and oncology treatment options. Early recognition and appropriate management of irAEs can significantly improve patients’ symptoms, prevent the cessation of potentially life-saving treatment, and enhance the quality of life of patients.

## Data Availability

The original contributions presented in the study are included in the article/Supplementary material, further inquiries can be directed to the corresponding author.
